# Optical painting and fluorescence activated sorting of single adherent cells labelled with photoswitchable Pdots

**DOI:** 10.1038/ncomms11468

**Published:** 2016-04-27

**Authors:** Chun-Ting Kuo, Alison M. Thompson, Maria Elena Gallina, Fangmao Ye, Eleanor S. Johnson, Wei Sun, Mengxia Zhao, Jiangbo Yu, I-Che Wu, Bryant Fujimoto, Christopher C. DuFort, Markus A. Carlson, Sunil R. Hingorani, Amy L. Paguirigan, Jerald P. Radich, Daniel T. Chiu

**Affiliations:** 1 Department of Chemistry, University of Washington, Seattle, Washington 98195, USA; 2 Clinical Research Division, Fred Hutchinson Cancer Research Center, Seattle, Washington 98109, USA

## Abstract

The efficient selection and isolation of individual cells of interest from a mixed population is desired in many biomedical and clinical applications. Here we show the concept of using photoswitchable semiconducting polymer dots (Pdots) as an optical ‘painting' tool, which enables the selection of certain adherent cells based on their fluorescence, and their spatial and morphological features, under a microscope. We first develop a Pdot that can switch between the bright (ON) and dark (OFF) states reversibly with a 150-fold contrast ratio on irradiation with ultraviolet or red light. With a focused 633-nm laser beam that acts as a ‘paintbrush' and the photoswitchable Pdots as the ‘paint', we select and ‘paint' individual Pdot-labelled adherent cells by turning on their fluorescence, then proceed to sort and recover the optically marked cells (with 90% recovery and near 100% purity), followed by genetic analysis.

## Abstract (web summary)

Isolation of individual cells from mixed populations is desirable for many biomedical applications. Here the authors use photoswitchable Pdots to allow 'optical painting', where cells of interest are marked based on their visual characteristics, and can then be isolated by fluorescence activated cell sorting.

## Graphical abstract

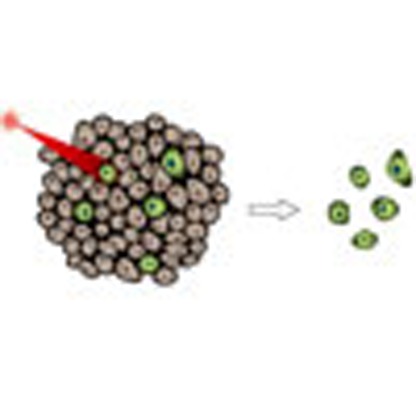

The efficient selection and isolation of cells of interest from a mixed population is important in biomedical research and biotechnology. Selected cells are often subjected to cell expansion^1^, transplantation^2^ and genetic analysis^3^. Expansion of selected cells is used to create various cell lines, such as cancer, stem and genetically engineered ones^4^,^5^. Transplantation of cells facilitates the establishment of tumour models in laboratory animals or the repair of damaged organs^2^. Analysis of specific cells in tissues contributes to the discovery of the biological interactions that drive diseases and aging^3^. All these applications would benefit from the ability to select, isolate and study individual cells in a high-throughput manner.

Various methods have been developed to select cells of interest according to their unique characteristics, such as morphology and biomarkers. These methods include fluorescence activated cell sorting (FACS), limiting dilution, cloning ring, panning, column chromatography and magnetic sorting^6^,^7^. Among these methods, FACS, in which individual cells of interest are sorted based on the presence of fluorescent probes that target cell-specific biomarkers, perhaps is the most popular and powerful approach because it offers high throughput and a lot of information with single-cell sensitivity^8^. A key constraint of FACS, however, is that when used to isolate cells from solid tumours or intact tissue, the adherent cells must first be separated by enzymatic dissociation and then downstream sorting relies on differences in the expression of cell markers^9^,^10^. Thus, FACS cannot be used to select and sort cells based solely on their morphology and location. This represents a potential limitation as the majority of cells in the body are adherent cells that grow and function in close contact with other neighbouring cells, such as in a tissue and most cells used in biomedical research are adherent cells grown on a culture plate and attached to a surface. Such spatial and morphological information can be critical for the investigation of cell behaviours in their microenvironment^11^. Palecek *et al*., for example, showed that the fate of stem cells can be determined in large part by their cell-to-cell contacts^12^, and Carlo and co-workers found cell division to be affected heavily by the degree of crowding in the space into which they are growing^13^.

Techniques have been developed to enable the selection and study of cells based on their spatial and morphological characteristics. Chief among these is laser capture microdissection that extracts selected target cells growing in a specific site of a thin tissue slice by physically cutting out the cell with a pulsed ultraviolet laser or by tearing out the desired cells by adhering them to a thin plastic membrane^14^,^15^. However, the extracting process potentially can damage or contaminate the target cells. Alternatively, Allbritton and co-workers and Lee *et al*. developed an array of micropallets for culturing and sorting adherent cells^1^,^3^,^4^,^5^. The cells cultured on the micropallets could be specifically removed with a laser pulse or a needle that mechanically displaces selected pallets from the array. Using magnetic pallets, individual pallets could be magnetically manipulated and deposited into a 96-well plate^16^. While this system is elegant and easy to use, it does require cells to be grown on a microfabricated array of pallets. For tissues, the adherent cells first would need to be dissociated and grown on the micropallets before selection and sorting.

As a result, there still is a need for a simple and high-throughput approach that enables individual adherent cells to be imaged in their native microenvironment, and then selected and isolated. This paper describes such an approach in which individual cells are optically ‘painted' during imaging so these marked cells may be isolated via FACS. First, we design and synthesize a new type of photoswitchable semiconducting polymer dots (Pdots), which can switch between the bright (ON) and dark (OFF) states reversibly. These photoswitchable Pdots have a 150-fold contrast ratio on irradiation with ultraviolet (for switch OFF) and red light (for switch ON). We then conjugate the photoswitchable Pdots to streptavidin and label cells via biotinylated primary antibodies. With a focused 633-nm laser beam, we can select and ‘paint' individual adherent cells under a microscope by turning on their fluorescence. We then proceed to sort and recover the optically marked cells by a home-built cell-sorting instrument we develop and which is capable of handling small sample volumes with minimal cell loss and high efficiency (90% recovery with near 100% purity), after which we also perform Sanger sequencing and messenger RNA (mRNA) extraction of the painted and sorted cells. Furthermore, we successfully apply this method for the painting and sorting of a mouse pancreas tumour tissue slice.

## Results

### Illustration of the optical painting and sorting method

**Figure 1:**
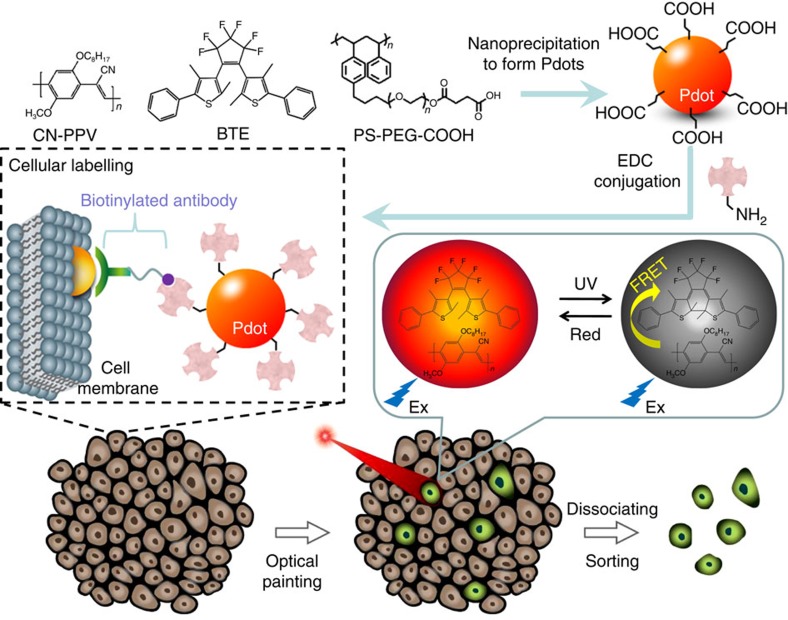
An overview of optical painting and fluorescence activated sorting of single adherent cells labelled with photoswitchable Pdots. Schematic depiction of Pdot formation, cellular labelling using Pdots and ‘painting' of labelled cells with light, followed by the sorting and isolation of painted cells.

Figure 1 schematically illustrates the strategy, where cells are first labelled with fluorescent probes that can be turned ON or OFF with light. After labelling, the cells may be studied by optical imaging. Cells of interest are optically marked with a focused laser beam for downstream FACS analysis and isolation. In this approach, the illuminating light is analogous to a paintbrush, and the photoswitchable fluorescent probes are akin to paint. How well this approach works relies heavily on the performance of the photoswitchable probes. To ensure the marked cells can be isolated with high efficiency and recovery using FACS, the photoswitchable probes must be bright and offer high contrast between the ON and OFF states so the selected cells can be easily detected and distinguished from all the other cells.

### Design of photoswitchable probes

The successful implementation of this strategy is highly dependent on the photoswitchable probes, which must have the following: (1) high brightness so the optically marked cells can be easily detected and recovered; (2) high contrast between the ON/OFF states so the marked cells can be accurately distinguished from background cells; (3) high-absorption cross-section so the labelled cells can be easily switched ON without requiring intense laser illumination; (4) good stability, especially thermostability, so the switched ON cells do not spontaneously decay back to the OFF state or vice versa; (5) a rapid conversion rate so individual cells can be painted in a high-throughput manner; (6) the capacity to be painted with red or near-infrared light to avoid cell damage and for increased penetration depth so cells buried deep in a biopsied tissue may be painted; (7) low photobleaching rate; and (8) good fatigue resistance.

On the basis of the above criteria, we surveyed various potential photoswitchable molecules. Candidates included different photochromic molecules^17^ and fluorescent proteins^18^,^19^,^20^. Photochromic molecules can be converted between isomers that exhibit different optical properties on light irradiation. However, the main optical difference between isomers is the absorption spectra, which cannot be used for fluorescence imaging. For fluorescent dyes that can be photoswitched between different states, such as those used for super-resolution microscopy, they are usually not sufficiently thermally stable with a back conversion time on the order of tens of minutes (for example, Cy5) (ref. 21). Photoactivable fluorescent proteins, in particular, are an attractive option because they provide high contrast. But they can be easily photobleached^18^,^19^,^20^. More critically, they require genetic manipulation of the cells of interest, which in some situations is highly desirable but in many others, not feasible (for example, biopsied samples) or even desirable. As a result, we decided to develop photoswitchable fluorescent probes that can be coupled with antibody labelling.

The platform on which we have chosen to develop this class of photoswitchable probes was based on semiconducting Pdots combined with a photochromic quencher. Pdots are a new family of ultra-bright fluorescent probes^22^. There are several compelling reasons for choosing Pdots: (1) they are extremely bright, up to 30 × brighter than even quantum dots and exceptionally photostable^23^; (2) they have fast photoemission rates, often with sub-nanosecond lifetimes so they are well suited for flow-based applications and FACS; (3) they possess good biocompatibility^24^,^25^ and are not composed of cytotoxic heavy metals such as quantum dots^26^; (4) most importantly, they exhibit amplified energy transfer so their fluorescence emission can be well modulated by photochromic molecules via energy transfer.

We have developed various classes of Pdots as ultra-bright probes for cellular imaging, flow cytometry and *in vivo* tumour targeting^23^,^26^. Among the different Pdots we have developed, poly[2-methoxy-5-(2-ethylhexyloxy)-1,4-(1-cyanovinylene-1,4-phenylene)] (CN-PPV) Pdots emit in the orange (590 nm) with a high quantum yield (60%) and have been applied successfully to cellular imaging and sorting^27^. Therefore, we chose CN-PPV Pdots as a model to construct the photoswitchable probe in this work.

For our design, we needed a photochromic quencher that can act as an energy acceptor and quench CN-PPV's fluorescence via Förster resonance energy transfer (FRET). The photo-induced conversion of the photochromic quencher controls the absence or presence of FRET, resulting in the ON–OFF switching of CN-PPV's fluorescence. Candidates of photochromic quenchers that we have studied and tested include azobenzenes, stilbenes, spiropyrans and diarylethene^17^.

For azobenzenes, they have a relatively low absorption cross-section and, more importantly, brightness contrast (4,000 and 2,000 M^−1^ cm^−1^ for the two isomers)^28^. Stilbenes suffer from irreversible cyclization and oxidation in the *cis*-form^29^. Spiropyrans are promising candidates; in fact, we have developed a photoswitchable Pdot based on spiropyrans for imaging applications^30^. Unfortunately, while they work for imaging, they cannot be used for photo-painting followed by FACS because they are not sufficiently thermally stable. After ultraviolet-induced conversion, they can thermally revert back to their original form before photoswitching within tens of minutes, offering insufficient time for FACS analysis.

Compared with these photochromic molecules, we found BTE (1,2-bis(2,4-dimethyl-5-phenyl-3-thienyl)-3,3,4,4,5,5,-hexafluoro-1-cyclopentene), a diarylethene, to offer excellent performance for our strategy. It has a high brightness contrast (∼10,000 M^−1^ cm^−1^ for quenched state but essentially transparent in the other state), good photoswitching kinetics (milliseconds), exceptional thermal stability (>90 days at 80 °C) and high fatigue resistance (cycled 10,000 times)^31^. Because of these desirable properties, BTE, in fact, has been used as a quencher for various fluorescent nanoparticles^32^,^33^,^34^,^35^. BTE has no quenching effect on Pdots in its initial open-ring state. However, after ultraviolet-induced photocyclization, the open-ring BTE transforms to the closed-ring isomer (Supplementary Fig. 1). The closed-ring isomer exhibits strong absorption bands (∼10,000 M^−1^ cm^−1^) in the visible range and can efficiently quench the fluorescence emitted from most fluorophores^31^,^36^. The closed-ring isomer is readily converted back to the open-ring form with red-light illumination, which turns on the fluorescence of the CN-PPV Pdot because its fluorescence is no longer quenched by the closed-ring form of BTE.

In our approach, as conceptually depicted in Fig. 1, the fluorescence emitted from CN-PPV Pdots was not affected by the open-ring BTE (ON state). But the Pdots' fluorescence was quenched by the closed-ring BTE (OFF state), which formed on ultraviolet irradiation. When the BTE-doped CN-PPV Pdots (CN-PPV-BTE Pdots) were irradiated by red light, the closed-ring BTE returned to its open-ring state, thereby turning ON again the fluorescence from CN-PPV Pdots.

### Characterization of CN-PPV-BTE Pdots

**Figure 2:**
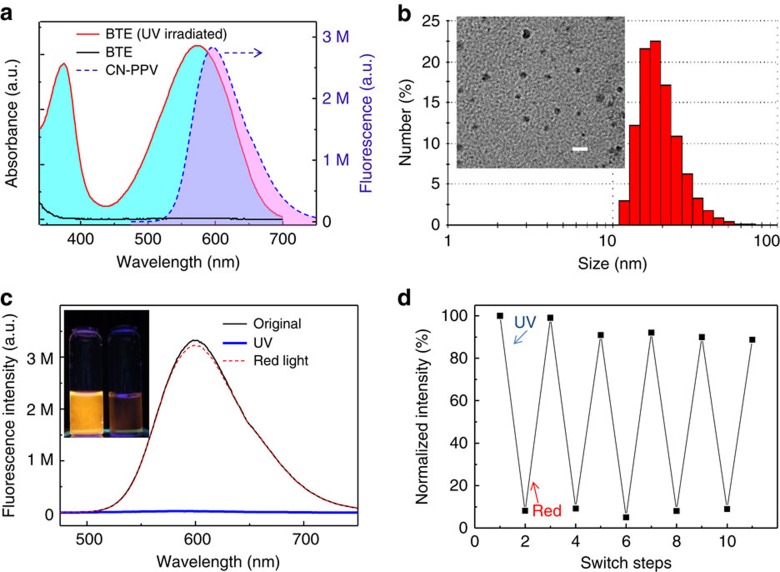
Characteristics of CN-PPV-BTE Pdots. (**a**) Ultraviolet (UV)–visible spectra of BTE with open-ring (black solid line) and closed-ring (UV irradiated, red solid line) states in THF and the fluorescence spectrum of CN-PPV Pdots in H_2_O (blue dash line, Ex 450 nm). (**b**) The size distribution of CN-PPV-BTE Pdots measured by the dynamic light scattering. The inset shows the transmission electron micrograph of Pdots. Scale bar, 50 nm. (**c**) The emission spectra (excitation at 450 nm) of the CN-PPV-BTE Pdots solution (black line, ON state), subsequently obtained after UV (blue line, OFF state) and red-light treatments (red dash line, ON state). The inset shows the photograph of Pdots solution at ON and OFF states. (**d**) The cyclic photoconversion of CN-PPV-BTE Pdots. ‘UV' and ‘red' indicate the exposure of samples under UV and red light. The intensity of fluorescence was recorded at 590 nm. The UV and red-light irradiations were achieved with a common hand-held UV lamp (254 nm, 310 μW cm^−2^) and a red light-emitting diode lamp (625 nm, 1.5 mW cm^−2^) for 20 s and 3 min, respectively.

Figure 2a shows the optical properties of BTE before and after ultraviolet irradiation. The open-ring BTE dissolved in tetrahydrofuran (THF) was transparent and had no absorption band in the visible wavelength region. After irradiation with ultraviolet light, the solution turned into a dark-blue colour and had two absorption bands centred at 375 and 580 nm. We relied on the absorption spectra of BTE dissolved in THF in our design of photoswitchable Pdots because the hydrophobic interior of Pdots is more similar to an organic phase, such as THF, than aqueous solution. In addition, BTE is insoluble in water. The absorption spectrum of the ultraviolet-irradiated BTE (closed ring) overlapped substantially with the emission spectrum of the CN-PPV Pdot in water centred at 590 nm (Fig. 2a), ensuring an efficient energy transfer from CN-PPV to the closed-ring BTE. Thus, the fluorescence emitted from CN-PPV can be quenched very efficiently by the closed-ring BTE via FRET.

The CN-PPV-BTE Pdots were prepared using a modified nanoprecipitation method. BTE, CN-PPV and an amphiphilic polystyrene (PS-PEG-COOH) were mixed and dissolved in THF and then injected into water under sonication. The formed Pdots were stable in water without aggregation. PS-PEG-COOH was used to functionalize Pdots with carboxyl groups for facile bioconjugation with streptavidin, which allows for specific cellular targeting via biotinylated primary antibodies. The size of the prepared Pdots was ∼20 nm in diameter as measured by the dynamic light scattering and transmission electron microscopy (Fig. 2b).

The optical characteristics and photoswitching performances of the CN-PPV-BTE Pdots were examined in bulk solution (Supplementary Fig. 2). Two absorption peaks at 270 and 450 nm from the CN-PPV-BTE Pdots (Supplementary Fig. 2b) were attributed to BTE and CN-PPV (Supplementary Fig. 2a), respectively. The optimized ratio by weight of BTE:CN-PPV:PS-PEG-COOH was 3:1:0.2. The corresponding doping ratio of BTE to CN-PPV monomer in Pdots was estimated to be 3:4 based on the molar extinction coefficients.

When the Pdots solution was irradiated with 254-nm light, the change in absorption spectrum (Supplementary Fig. 2b) indicated the conversion of doped BTE from the open-ring to the closed-ring form. After irradiation of the bulk sample with red light (625 nm), the absorption spectrum recovered, indicating the regeneration of the open-ring BTE.

Figure 2c shows the reversible photoswitching of CN-PPV-BTE Pdots (in water) in a single cycle of illumination with ultraviolet and red lights. The fluorescence of Pdots significantly decreased (OFF state) on irradiation with ultraviolet light and recovered (ON state) after illumination with red light. The fluorescence intensity ratio of Pdots at 590 nm between the ON state and the OFF state, that is, ON/OFF ratio, was 150. Compared with our previous photoswitchable Pdots composed by poly[(9,9-dioctylfluorenyl-2,7-diyl)-co-(1,4-benzo-{2,1′,3}-thiadiazole)] (PFBT) and spiropyran molecules^30^, the ON/OFF contrast ratio was improved 20 times. The fluorescence quantum yield of Pdots in the ON state was measured to be ∼51% and decreased to ∼0.4% in the OFF state.

After repeated cycles of photoswitching, we did observe a slight decay of fluorescence from Pdots, which may be caused by photobleaching. To investigate this, CN-PPV Pdots without doped BTE and CN-PPV-BTE Pdots were examined under the same ultraviolet illumination conditions (Supplementary Fig. 2c). Under these conditions, the fluorescence of the CN-PPV Pdots decreased to 95% of its original value after ∼20 s of exposure to ultraviolet light. In contrast, the fluorescence of CN-PPV-BTE Pdots markedly decayed to ∼1% within 20 s of ultraviolet irradiation because of FRET. The fluorescence of CN-PPV-BTE Pdots was recovered with red-light irradiation. From these results, we confirmed that the marked decay of CN-PPV-BTE Pdots' fluorescence after ultraviolet irradiation was dominated by FRET and was not caused by photobleaching. The decrease of fluorescence lifetime of CN-PPV-BTE from 1.14 to 0.75 ns after irradiation with ultraviolet light (Supplementary Fig. 3) also verified the presence of FRET. Twenty seconds appear sufficient to fully convert the open-ring to the closed-ring form of BTE, and thus we chose 20 s as the period of ultraviolet irradiation in all of our experiments to minimize any potential for photobleaching. Red-light illumination did not cause any photobleaching of the Pdots (Supplementary Fig. 2c) so the duration of red-light irradiation could be extended to several minutes until the fluorescence signal stopped to increase with time (that is, completely recovered).

The reversibility and reproducibility of photoswitching of CN-PPV-BTE Pdots was assessed using repeated cycles of ultraviolet and red-light irradiations. In Fig. 2d, the Pdots were switched reversibly between OFF and ON states for five cycles, which exhibited excellent fatigue resistance and indicated that the BTE molecules in the Pdots were stable throughout the photoswitching cycles. After several cycles of photoswitching, we observed a slight decay of fluorescence from Pdots, which was caused by photobleaching. Supplementary Figure 2d shows the excellent thermal and colloidal stability of the photoswitchable Pdots: the OFF state persisted at room temperature for >1 week before we returned the Pdots to the ON state with red-light irradiation.

To investigate the dependence of the photoswitching efficiency on the amount of BTE, we recorded the fluorescence quenching efficiency (defined as 1−(OFF/ON ratio)) and the doping ratio (mole ratio of BTE to CN-PPV monomer, determined from the molar extinction coefficients) as a function of the feeding ratio of BTE to CN-PPV (by weight). There was a positive correlation between the feeding ratio and the quenching efficiency as well as the doping ratio (Supplementary Fig. 4). When the feeding ratio was over 3:1 (BTE: CN-PPV), the quenching efficiency reached a saturated level even when the doping ratio continued to increase.

The amounts of PS-PEG-COOH blended in Pdots were shown to have no effect on the photoswitching efficiency (Supplementary Fig. 5). We used a blending ratio of 20% (by weight) PS-PEG-COOH to CN-PPV, which was the ratio we often used in our past experiments for bioconjugation of biomolecules to Pdots.

### Photoswitching performance of cells labelled with Pdots

To turn cells ‘ON' and make them fluorescent with red-light irradiation, we labelled the surface of MCF-7 cells with CN-PPV-BTE-streptavidin (CN-PPV-BTE-SA) Pdots using a biotinylated primary antibody against Epithelial cell adhesion molecule (EpCAM). EpCAM is the epithelial cell surface marker used for the isolation of tumour cells that exhibit epithelial characteristics. To quantify the photoswitching performance of Pdot-labelled cells, we used flow cytometry.

**Figure 3:**
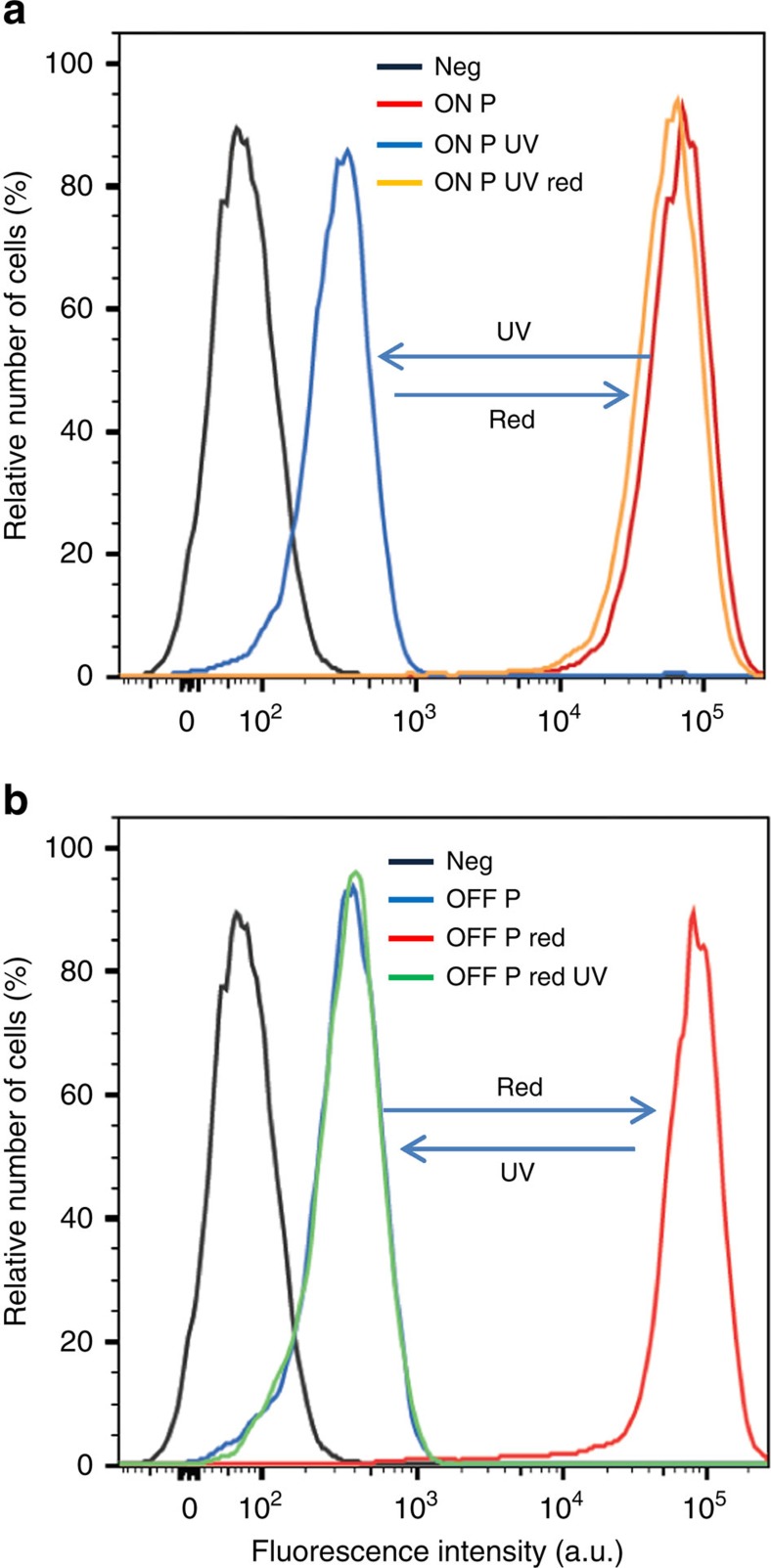
The reversible photoswitching of cells in bulk as measured by flow cytometry. Whether the MCF-7 cells were initially labelled with CN-PPV-BTE Pdots at (**a**) ‘ON' state or (**b**) ‘OFF' state, the fluorescence intensity distributions of cells reversibly changed upon irradiation of ultraviolet (UV) and red light. The negative control set of cells (Neg) that were not labelled with Pdots showed the lowest fluorescence intensity. The UV treatment was carried out by a hand-held UV light (254 nm, 310 μW cm^−2^) for 20 s; the red-light treatment was carried out with a red light-emitting diode (625 nm, 1.5 mW cm^−2^) for 3 min. The fluorescence signal from the flow cytometry was collected in the PE channel (that is, with 556 nm long pass and 585/42 nm band pass) on excitation at 488 nm.

As shown in Fig. 3, Pdot-labelled cells exhibited reversible ON–OFF switching on irradiation with ultraviolet and red light regardless of what the initial state the Pdots were in. The fluorescence intensity distributions of OFF-state and ON-state cells were well resolved from each other (Fig. 3). Specifically, the fluorescence peak of the ON-state cells was around 150 times brighter than that of the OFF-state cells in the flow cytometry measurement, which is more than sufficient for reliable FACS isolation of the ON cells. For the negative control (Neg), cells were incubated with CN-PPV-BTE-SA Pdots (ON state) but without biotinylated primary antibodies. The very low fluorescence of these negative control cells indicated an extremely low nonspecific binding of the Pdots to the cells. To prevent potential cell damage by ultraviolet irradiation during the photoswitching process, we first turned all Pdots to the OFF state before labelling the cells. We then turned the Pdot-labelled cells ‘ON' via irradiation with the more cell-friendly red light.

### Painting individual adherent cells

**Figure 4:**
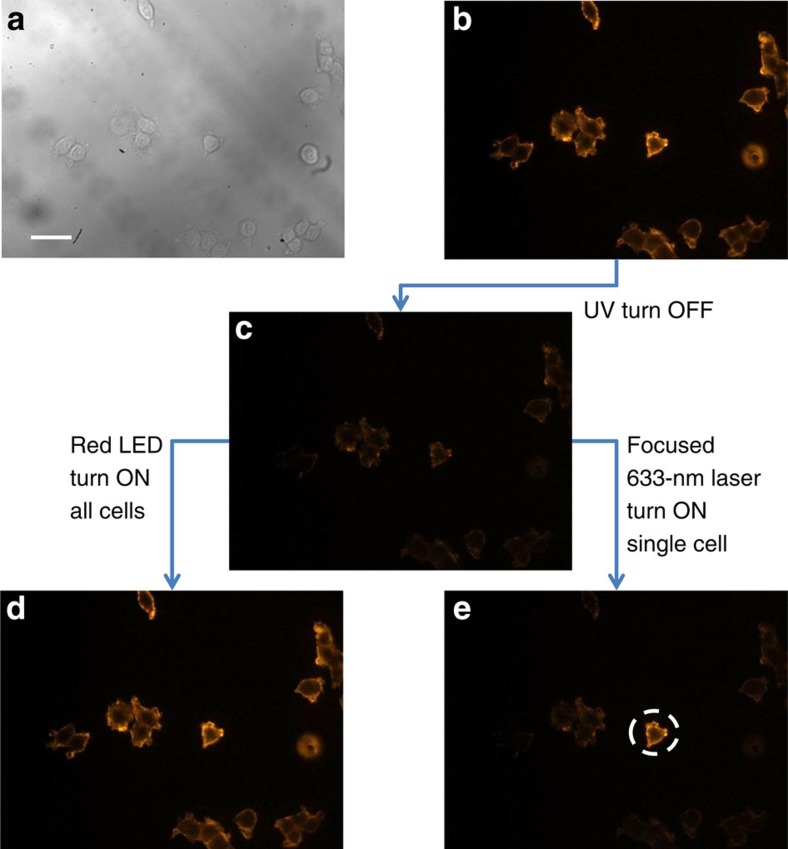
Selectively painting individual MCF-7 cells with a 633-nm laser beam. (**a**) A bright-field image of cells. Scale bar, 50 μm. (**b**–**e**) Fluorescence images showing the painting of (**d**) all cells or (**e**) a single cell within a population. The cell exposed to the focused 633-nm laser turned bright (dashed circle), while the other cells stayed dark. The ultraviolet (UV) treatment was carried out by a hand-held UV lamp (254 nm, 310 μW cm^−2^) for 20 s. The single-cell painting was achieved by a focused 633-nm laser (∼70 μW or 90 W cm^−2^) for 10 s; the turning ON of all cells was accomplished by wide-area illumination using a red light-emitting diode light (625 nm, 1.5 mW cm^−2^) for 3 min. The fluorescence signal was collected above 500 nm on excitation at 488 nm.

To demonstrate our optical painting approach (Fig. 1), we labelled MCF-7 cells grown on a Petri dish with photoswitchable CN-PPV-BTE-SA Pdots bound to biotinylated anti-EpCAM antibodies. From a practical perspective, cells were generally labelled first with OFF-state Pdots, which were formed by irradiating a bulk Pdot sample with ultraviolet light before cellular labelling. This way, we prevented cells from being directly irradiated with ultraviolet light, which has the potential to cause cellular damage. To turn on the OFF-state Pdot-labelled cells, we used cell-friendly red light to turn on individual cells, which we imaged and selected under a microscope. In Fig. 4, however, we used cells labelled with ON-state Pdots to demonstrate a complete photoswitching cycle. The ON cells appeared orange because of the 590-nm emission from CN-PPV (Fig. 4b). However, fluorescence was not observed from the negative control cells where cells were incubated with CN-PPV-BTE-SA Pdots but in the absence of biotinylated primary antibodies (Supplementary Fig. 6), again indicating the absence of nonspecific binding. Consistent with the results of flow cytometry (Fig. 3), the CN-PPV-BTE-SA Pdot-labelled cells became non-fluorescent after illumination with ultraviolet light (Fig. 4c); after red light-emitting diode irradiation, they recovered their orange fluorescence (Fig. 4d).

To achieve high-precision and high-throughput single-cell painting under a microscope, we used a focused 633-nm laser spot of 10 μm in diameter to match the dimension of a cell. Figure 4e shows the exposed cell changed from dark to bright (dashed circle); the other cells that were not illuminated by the focused 633-nm light remained dark. By tuning the scanning range or the spot size of the focused 633-nm laser beam, we carried out the painting of multiple cells and even a portion of a single cell (Supplementary Fig. 7). These results show the versatility of the optical painting method for a range of potential applications.

### Optical painting of cells with selective photobleaching

In addition to using photoswitching for cell painting, we have explored other methods of marking cells. One simple strategy is to label cells with two different fluorescent probes exhibiting different colours and then to bleach one of the two colours to mark the selected cell. In this scheme, the unmarked cell emits fluorescence in both colours, but the optically marked cell emits fluorescence only in one colour. This approach has the benefit that most existing conventional dyes may be used without the need of developing a new photoswitchable probe. Here we examined the feasibility and the practicality of this method and compared it with optical painting with photoswitchable Pdots.

In general, photobleaching is viewed as a drawback^37^. But it has been exploited for studying the dynamics of fluorescent proteins in live cells^38^. In our experiments, we labelled MCF-7 cells with a green fluorescent dye and a red fluorescent dye, with both conjugated to anti-EpCAM antibody. Then, we used a focused 633-nm laser beam to bleach the red dye (Supplementary Fig. 8a). For the green–red dye pairs, we tested fluorescein isothiocyanate (FITC)/APC, Alexa 488/APC and Alexa 488/Alexa 647. For the imaging and photobleaching, we used a commercial confocal microscope (Zeiss LSM 510, Jena, Germany). Supplementary Figure 8b–d show our results: after irradiation with the 633-nm laser light for several minutes: the red dyes (APC and Alexa 647) were bleached while the green dyes (FITC and Alexa 488) remained bright and gave off the green fluorescence only from the marked cells.

While our results suggest photobleaching represents an alternative approach for cell painting, we did notice several practical drawbacks compared with photoswitching: (1) the laser power required for photobleaching was higher than that needed for photoswitching, which may cause cell stress; (2) the time required for photobleaching was much longer than that necessary for photoswitching, which reduces throughput; (3) unlike photoswitching, photobleaching is not reversible; and (4) most critically, we found the contrast ratio offered by photobleaching (∼5) was much lower than that of optical painting with photoswitchable Pdots (∼150); the contrast of photobleaching was often insufficient to achieve high cell recovery with downstream FACS. Nevertheless, photobleaching represents a potential alternative to cell painting and offers the simplicity in that no new probe development is necessary.

### Cell sorting

To collect the painted cells by FACS, we used a home-built platform we called eDAR (ensemble-decision aliquot ranking). eDAR was designed and demonstrated to have high recovery efficiency (95%) for the isolation of rare cells from whole blood. It offers high sensitivity and high throughput: the method can process 1 ml of whole blood containing ∼5 billion red and white blood cells within 20 min (refs 39, 40). Compared with a commercial FACS instrument, which often requires large amounts of sample cells entering it (10^6^–10^7^) (ref. 1), eDAR is more flexible in that it can process both large sample volumes (several millilitres of whole blood) with a large number of cells (10^9^–10^11^) and small sample volumes (few microliters) with limited cell numbers (10–10^3^) (refs 39, 40).

**Figure 5:**
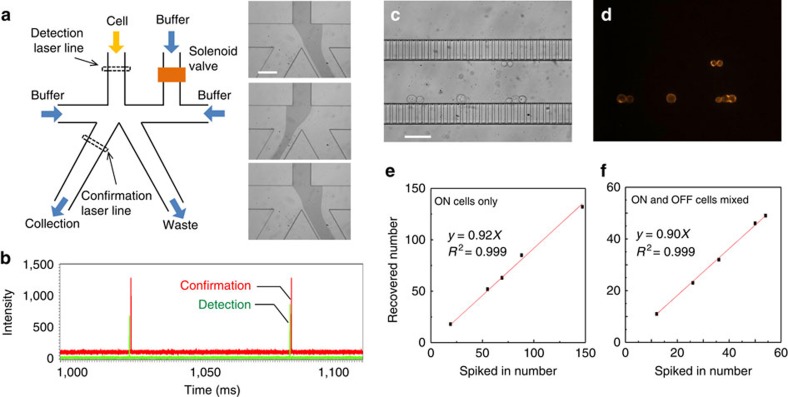
Cell-sorting process by a home-made cell-sorting system called ensemble-decision aliquot ranking (eDAR). (**a**) A simple schematic illustrating the eDAR concept. The insets show the flow being switched to the collecting filter and then switched back shortly later. A food dye was added to visualize the streamline. Scale bar, 50 μm. (**b**) A representative segment of the fluorescence traces recorded by the detection avalanche photodiode detector (APD; green) and the confirmation APD (red). The sorted cells exhibited fluorescence peaks in both channels. (**c**) The bright-field and (**d**) fluorescence images of the collected cells in the filters with a 5-μm height and 5-μm width. Scale bar, 50 μm. The fluorescence image was obtained by excitation via a green light from a Xe lamp and by collecting the fluorescence with a 570/20-nm band-pass filter. The recovery ratio of picking the ON-state MCF-7 cells by eDAR from (**e**) a pure sample solution and with (**f**) a population of around 300 OFF-state MCF-7 cells.

Figure 5a is a schematic of the eDAR microfluidic chip in which the fluorescence of cells was excited by line-confocal laser excitation and the resulting fluorescence signal was detected by an avalanche photodiode detector. When the detected fluorescence of a cell was above a set threshold, the solenoid valve was triggered to open and deflect the main-channel flow from the default waste channel into the cell-collection channel (Fig. 5a, insets).

Located in the collection channel and immediately after the sorting junction was a second line-confocal detection region, which confirmed successful cell sorting in real time (Fig. 5b). An integrated on-chip filter was located at the end of the cell-collection channel to retain the sorted cell for downstream imaging and analysis (Fig. 5c,d). Non-fluorescent cells that did not trigger sorting just flowed through to the waste channel by default and were not collected.

Using the eDAR system, we investigated the recovery efficiency of the ON-state cells and determined it to be 92% (Fig. 5e). To ensure eDAR could distinguish the ON-state from the OFF-state cells, we introduced these two types of cells through the eDAR device separately and analysed their respective fluorescence spike intensities (Supplementary Fig. 9a,b). Finally, we mixed these two types of cells together and introduced them through eDAR to investigate the recovery efficiency of the ON-state cells in a background of OFF-state cells. Supplementary Figure 9c shows a representative portion of the recorded fluorescence intensity trace, where five peaks were detected in the trace. However, four of the five peaks had a low fluorescence intensity and were below the threshold for sorting; these peaks were from OFF-state cells. Only one peak had a high fluorescence intensity above the sorting threshold; this peak was from an ON-state cell. In this experiment, to mark the OFF-state cells, we labelled them with a red dye (Alexa 647). In this mixed ON-state and OFF-state cell sample, the recovery efficiency was 90% (Fig. 5f).

On the basis of our flow cytometry analysis (Fig. 3), there may be a small overlap in the fluorescence distribution between ON-state and OFF-state cells, which potentially may result in false positives, that is, the sorting and collection of an OFF-state cell. This false positive would not reduce the recovery efficiency, but decrease the purity of the collected ON-state cells. To determine the false-positive rate, the OFF-state cells were labelled with Alexa 647-tagged antibody against cytokeratin before being mixed with ON-state cells. This way, OFF-state cells could be clearly distinguished from ON-state cells. In this experiment, we did not find any OFF-state cells within the population of ∼50 collected cells (Supplementary Fig. 10), thus indicating a near zero false-positive rate and a high purity in the collection of painted cells. The photo-painting and cell-sorting process is also applicable to live cells. Using live MCF-7 cells, we found that the viability was 90±5% after the entire process of Pdot labelling, photo-painting, trypsinization and cell detachment, and eDAR sorting and recovery (Supplementary Fig. 11). The recovery is also as high as that obtained with fixed cells.

### DNA sequencing and mRNA extraction

To examine whether the optical painting/sorting method is compatible with downstream analysis, the painted and sorted cells were subjected to either DNA sequencing (Sanger sequencing) or mRNA analysis. Information obtained from DNA sequencing or mRNA expression can indicate patient prognosis and inform treatment decisions. Typical genetic analysis methods require thousands to millions of cells for sufficient nucleic acid to be isolated for downstream analysis. Because of either tumour heterogeneity or the presence of healthy tissue in a biopsy, these samples can include a minority population of the cells of interest. The presence of the cells of interest in low concentrations can result in the minority molecular feature to be below the limit of detection via many techniques^41^. Using the painting/sorting method presented here, cells identified to be of interest can be obtained in high purity for genetic analysis.

**Figure 6:**
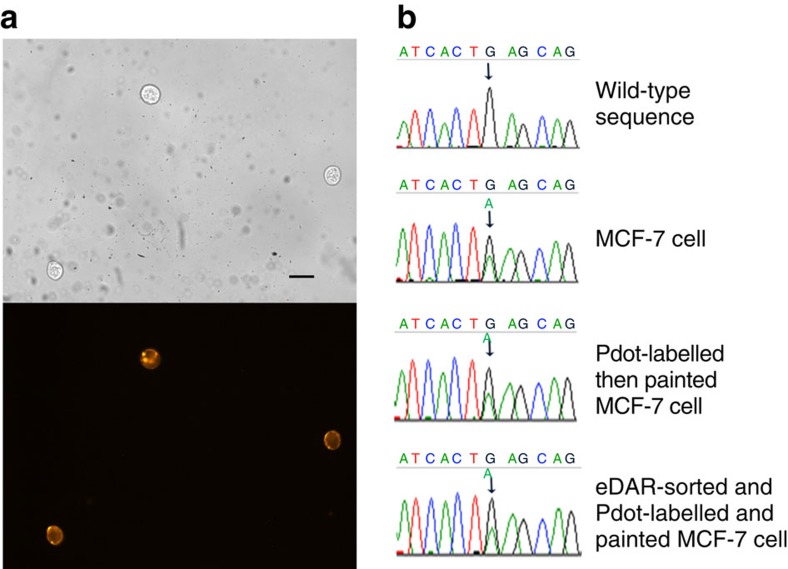
DNA sequencing of cells collected through the optical painting and sorting process. (**a**) Cellular image of eDAR-sorted cells. The fluorescence image was obtained by excitation via a green light from a Xe lamp and by collecting the fluorescence with a 570/20-nm band-pass filter. Scale bar, 50 μm. (**b**) Sequencing reads for exon 9 of the *PIK3CA* gene. A sequence variant c.1633 G>A (as indicated by arrows) was detectable from PCR products generated from MCF-7 cells not labelled with Pdots (‘MCF-7 cell'), after Pdot labelling and cell painting (‘Pdot-labelled then painted MCF-7 cell') and after eDAR sorting of the painted and Pdot-labelled cells (‘eDAR-sorted and Pdot-labelled and painted MCF-7 cell'). The wild-type sequence reads only G at *PIK3CA* c.1633.

Herein, we demonstrate the ability to sequence a portion of the *PIK3CA* oncogene as an example downstream analysis following this painting and sorting method. This gene is commonly mutated in breast and colorectal cancers^42^ and can confer increased cell survival, proliferation and migration^43^. The MCF-7 cell line is known to carry a heterozygous mutation c.1633 G>A in the *PIK3CA* gene^44^. In Fig. 6, as expected, this mutation was detected in both native and Pdot-labelled MCF-7 cells (after optical painting) as well as MCF-7 cells that underwent labelling, painting, detachment and eDAR sorting. In contrast, the wild-type sequence reads only G at *PIK3CA* c.1633. This result shows that both the painting and sorting process did not affect the interpretation of mutant status and genetic information of the cells, and that the optical painting and sorting method is compatible with downstream analysis.

To determine the impact of the optical painting/sorting procedures on mRNA quantity and quality, an mRNA integrity experiment was performed (Supplementary Fig. 12). For this experiment, MCF-7 cells went through the process of Pdot labelling, optical painting and eDAR sorting. The sorted cells were flowed into an exit channel/tubing placed after the sorting junction of the eDAR chip, and then collected in an Eppendorf tube for the subsequent mRNA extraction. Control sets including untreated and Pdot-labelled MCF-7 cells were examined in parallel to investigate the effect of Pdot labelling on mRNA recovery. The quantity of recovered RNA per cell relative to untreated cells was found to be 93.8 and 90.1% for the Pdot-labelled cells and painted then eDAR-sorted cells, respectively. In addition, the isolated RNA was of high quality for all samples, with an RNA Integrity Number (RIN^e^) of 9.6, 9.6 and 9.9 for untreated cells, Pdot-labelled cells and Pdot-labelled then painted and eDAR-sorted cells, respectively (RIN^e^ is reported on a 1–10 scale, with 10 indicating the highest quality). The results show that there is no significant mRNA damage caused by the Pdot labelling process nor by the optical painting and eDAR-sorting steps, thereby indicating the high compatibility of our optical painting method with downstream analysis involving gene sequencing and mRNA expression measurements.

### Application of optical painting to tissue slices

To demonstrate the relevance and potential applications of our optical painting technique in the context of tissues, we transitioned from cultured cell lines to murine pancreas tumours using a well-established genetically engineered mouse model of pancreatic ductal adenocarcinoma (PDA)^45^. PDA is among the most lethal of human malignancies and much effort has been spent on developing methods for early detection and on exploring mechanisms of pathogenesis in this cancer. In our experiment, a ∼50-μm-thick tissue slice from a mouse pancreas tumour was incubated with biotinylated anti-mouse EpCAM antibody, followed by CN-PPV-BTE-streptavidin (CN-PPV-BTE-SA) Pdots. The Pdot-labelled tissue showed bright-orange fluorescence under a microscope but dim fluorescence from the negative control, which was incubated with CN-PPV-BTE-SA Pdots but in the absence of biotinylated primary antibodies (Supplementary Fig. 13). This result indicates the efficient labelling of the tissue by Pdots and the presence of minimal nonspecific binding.

**Figure 7:**
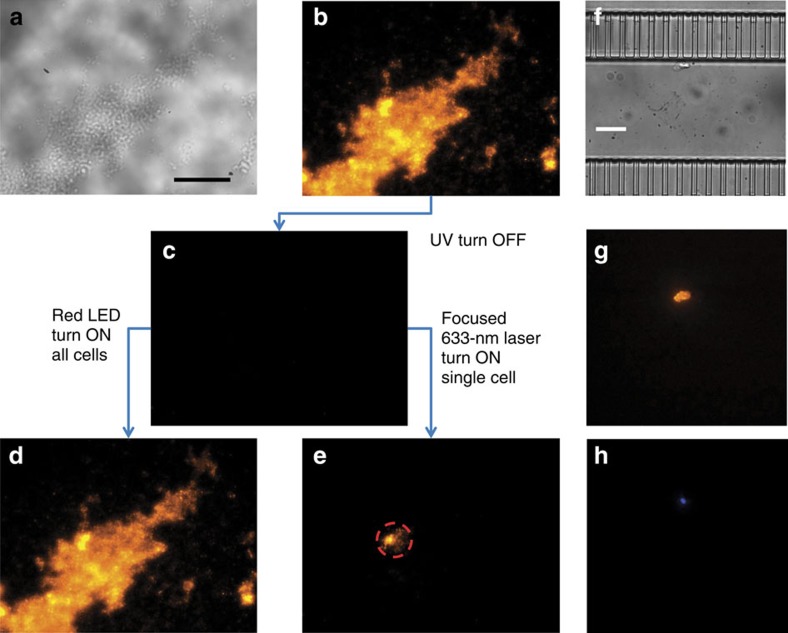
Optical painting and eDAR recovery of painted cells from a mouse pancreas tumour tissue slice. (**a**,**b**) A bright-field and fluorescence image of the tissue slice labelled with photoswitchable Pdots. (**c**) Application of ultraviolet (UV) light turned OFF the fluorescence of all cells in the tissue. (**d**,**e**) Fluorescence images showing the (**d**) photoswitching ON of the fluorescence of all tissue cells with a red light-emitting diode (LED) and wide-field illumination, and (**e**) selectively painting of individual tissue cells with a focused 633-nm laser beam, where the illuminated cell turned bright (dashed circle), while the other cells remained dark. The painted tissue cells could be sorted and recovered by the eDAR system, and then observed under (**f**) bright field and (**g**,**h**) fluorescence; (**g**) CN-PPV Pdot fluorescence and (**h**) the nuclear stain, DAPI, fluorescence. The ‘UV turn-OFF' treatment was carried out using a hand-held UV lamp (254 nm, 310 μW cm^−2^) for 20 s. The selectively painting was achieved by illumination with a focused 633-nm laser (∼70 μW or 90 W cm^−2^) for 1 min; the turning ON of all tissue cells was accomplished by wide-area illumination using a red LED light (625 nm, 1.5 mW cm^−2^) for 5 min. The fluorescence signal of (**b**–**e**) was collected with a 500-nm long-pass filter on excitation at 488 nm. The fluorescence signal of **g** and **h** was obtained by excitation via a Xe lamp and by collecting the fluorescence through a 570/20-nm (for CN-PPV fluorescence) and a 460/30-nm (for DAPI fluorescence) band-pass filter, respectively. Scale bar, 20 μm.

The Pdot-labelled tissue successfully exhibited photoswitching and could be selectively painted (Fig. 7a–e), similar to the painting of MCF-7 cells shown in Fig. 4. With a focused 633-nm laser, we could control the painting area and the number of cells to be painted (Fig. 7e). Furthermore, the painted cell could be recovered as shown in Fig. 7f–h, where a representative cell (∼10 μm) in the tissue slice was painted, after which the tissue was dissociated and the painted cell was recovered by the eDAR system. Here all cells of a tissue slice would be dissociated but only the optically painted bright cells would be isolated by the eDAR system (Fig. 7g). We labelled the tissue with 4,6-diamidino-2-phenylindole (DAPI) before dissociation (Supplementary Fig. 13) so that we could confirm the collected cells were intact based on the DAPI signal from cell nucleus (Fig. 7h). The successful application of optical painting to tissue slices demonstrates the utility of this method for studying adherent cells in a tissue.

## Discussion

This study demonstrates a new concept for sorting adherent cells. Individual cells of interest are optically ‘painted' during imaging so that these cells with their fluorescence turned on can be analysed with flow cytometry or isolated by FACS. This approach is inherently compatible with imaging and offers high throughput because of FACS' fast cell-sorting speed. To implement this concept, we developed photoswitchable Pdots that were exceptionally bright, provided high brightness contrast, were reversible with good photoswitching fatigue resistance, had fast photoswitching kinetics and were thermally stable and did not spontaneously revert back to the dark state or vice versa. In addition, these photoswitchable Pdots were turned on with red light that exerts less cell stress than ultraviolet or blue light. Finally, the painted cells were recovered with high efficiency and excellent purity. The throughput of this cell painting and sorting method is limited fundamentally by the time it takes to paint the cells as well as the subsequent cell sorting. For sorting, we have shown that eDAR has a sorting speed on the order of milliseconds^39^. For cell painting, the laser irradiation time is also on the order of milliseconds for a focused laser spot with sufficient power. In addition, the laser spot may be enlarged to paint multiple cells simultaneously. As a result, the current throughput limitation is caused by the need for manual cell imaging, decision making and photo-painting. If an automated system is developed in which cells are marked automatically based on some pre-defined parameter, this approach has the potential to achieve high throughput.

Compared with conventional immuno-fluorescence staining method, our approach allows cells to be selected based on spatial and morphological or microenvironmental information. In some sense, the cells of interest are ‘sorted' or selected during the photo-painting process, and subsequent trypsinization and FACS is just a means of recovering those optically marked cells.

With further optimization, the performance of the photoswitchable Pdots and the recovery efficiency may be further improved. As a result, we believe this method extends the power of FACS to adherent cells and allows single adherent cells to be sorted and isolated based on their morphology and spatial location in a cellular network. Furthermore, this cell photo-painting technique may be used to mark cells on the basis of any desired dynamic properties, such as cell division rates, cell migration, differentiation, behavioural phenotypes, response to drugs and so on. This capability will enhance the study of single cells within tissues and should be useful for both basic biological studies and clinical research.

## Methods

### Materials

All of the chemicals and solvents were purchased from Sigma-Aldrich (St Louis, MO, USA) unless indicated otherwise. The fluorescent semiconducting polymer CN-PPV (molecular weight (MW) 15,000, polydispersity 5.9) was purchased from ADS Dyes, Inc. (Quebec, Canada). The photochromic quencher BTE was purchased from TCI (Portland, OR, USA). A comb-like polymer—polystyrene grafted with ethylene oxide functionalized with carboxyl groups (PS-PEG-COOH, main chain MW 8,500, graft chain MW 1,200, total chain MW 21,700, polydispersity 1.25)—was purchased from Polymer Source Inc. (Quebec, Canada). The fluorochromes, including FITC, APC, Alexa 488 and Alexa 647, used in the photobleaching experiment were purchased from BD Bioscience (San Jose, CA, USA). HEPES, EDC (1-ethyl-3-[3-dimethylaminopropyl]carbodiimide hydrochloride) and streptavidin were purchased from Invitrogen (Eugene, OR, USA). All chemicals were used as received without further purification. High purity of MilliQ water (18.2 MΩ  cm^−1^) was used throughout the experiment.

### Preparation of photoswitchable CN-PPV-BTE Pdots

The photoswitchable Pdots were prepared using a modified nanoprecipitation method. Typically, CN-PPV, BTE and PS-PEG-COOH were separately dissolved in THF (anhydrous, ≥99.9%, inhibitor free) to make three 1 mg ml^−1^ stock solutions. PS-PEG-COOH was blended with Pdots to functionalize the carboxyl groups for bioconjugation. Suitable amounts of CN-PPV, BTE and PS-PEG-COOH stock solution were taken to get the desired concentrations and doping ratios. Typically, 0.1 ml CN-PPV (1 mg ml^−1^), 0.3 ml BTE (1 mg ml^−1^) and 0.02 ml PS-PEG-COOH (1 mg ml^−1^) were mixed. The mixture was sonicated to form a homogeneous solution and then quickly injected to 10 ml MilliQ pure water in a bath sonicator for 30 s. Then, the THF was removed by nitrogen stripping and heating at 90 °C. Finally, the residue solution was passed through a 0.2-μm filter from VWR (Radnor, PA, USA) to remove the aggregation particles and precipitations, such as the undoped BTE. The synthesized CN-PPV-BTE Pdots were well dispersed in water and stable for months without aggregation.

### Characterization of Pdots

Ultraviolet/visible absorption spectra of the Pdots solution were recorded with a DU 720 scanning spectrophotometer from Beckman Coulter, Inc. (Brea, CA, USA). Fluorescence spectra were acquired in a commercial Fluorolog-3 fluorometer from HORIBA Jobin Yvon Inc. (Edison, NJ, USA). Fluorescence lifetime measurements were carried out using a PicoQuant Fluorescence lifetime system (PicoQuant Photonics North America Inc., Westfield, MA, USA). The particle sizes of Pdots in bulk solution were measured by the dynamic light scattering (NanoS Zetasizer) from Malvern (Westborough, MA, USA). For the electron micrograph measurements, two to three drops of the Pdots solution (∼20 p.p.m.) were placed on a carbon-coated copper grid (200 mesh) provided from TED PELLA, Inc. (Redding, CA, USA) and then dried under ambient conditions. The dried Pdots' sizes were measured with a transmission electron microscope (Tecnai F20) from FEI (Hillsboro, OR, USA).

### Bioconjugation of Pdots

Bioconjugation was carried out by coupling carboxylate-functionalized Pdots and the amine-containing streptavidin purchased from Invitrogen via EDC-catalysed coupling. Typically, for a 4-ml Pdot solution with the concentration of 50 p.p.m., 80 μl polyethylene glycol (5% w/v PEG, MW 3,350) and 80 μl HEPES buffer (1 M, pH 7.3) were mixed. Then, 240 μl streptavidin (1 mg ml^−1^ in 20 mM HEPES buffer) was added to the Pdots solution and mixed on a vortex. Finally, 80 μl of freshly prepared EDC (10 mg ml^−1^ in MilliQ water) was added to the Pdots solution. The mixture was stirred for 4 h at room temperature. After bioconjugation, 80 μl bovine serum albumin (BSA; 10 wt %) was added to the Pdots solution, and the reaction was continued for another 20 min to eliminate the aggregation of Pdots. An 80 μl aliquot of Triton X-100 (2.5 wt % in MilliQ water) was added to the Pdots solution to make the Pdots more stable. The mixture then was transferred to a centrifuge ultrafiltration tube (Amicon Ultra-4, molecular weight cutoff: 100 kDa, from EMD Millipore, Billerica, MA, USA) and then concentrated to 0.5 ml by centrifugation. Finally, the mixture was purified by gel filtration via Sephacryl HR-300 gel media to obtain streptavidin-functionalized Pdots for cellular labelling. Aliquots of 50 μl BSA (10 wt %) and 5 μl sodium azide were added to the purified Pdots solution for long-term storage.

### Cell culturing

The breast cancer cell line MCF-7 cell and cell culture medium were purchased from American Type Culture Collection (Manassas, VA, USA). The MCF-7 cells were cultured at 37 °C with 5% CO_2_ in Eagle's minimum essential medium (with L-glutamine) supplemented with 10% fetal bovine serum and 1% penicillin (50 U ml^−1^)–streptomycin (5 μg ml^−1^) solution. The cells were pre-cultured in culture flasks until ∼80% confluency was reached.

### Cell labelling for flow cytometry

To collect the cells, the adherent cells were quickly rinsed with media and then incubated in 5 ml trypsin-EDTA solution (0.25 w/v % trypsin, 2.5 g l^−1^ EDTA) at 37 °C for 5–10 min to suspend the cells. The detached cells were collected in a tube and then centrifuged at 700*g* for 10 min to precipitate them to the bottom of the tube. After removing the upper media, the cells were rinsed and resuspended in 5 ml culture media. The cell concentration was determined under the microscope with a hemocytometer.

For cellular labelling experiments, ∼10^6^ cells were transferred to 100-μl labelling buffer (1 × PBS, 2 mM EDTA, 1% BSA) and incubated with 0.6 μl of 0.5 mg ml^−1^ primary biotinylated anti-human CD326 (EpCAM) antibody purchased from BioLegend (San Diego, CA, USA, cat. #324216) on a rotary shaker in the dark and at room temperature for 30 min. This was followed by a washing step using labelling buffer to remove the excess antibody. For the conjugation of cells and Pdots, the biotinylated cells were incubated with ∼5.0 nM streptavidin-functionalized Pdots in 0.2 ml BlockAid buffer purchased from Invitrogen for 30 min on a rotary shaker in the dark and at room temperature, followed by two washing steps with labelling buffer to remove the excess Pdots. The streptavidin-functionalized Pdots solution was sonicated for 3 min to disperse any potential aggregation. Negative controls were obtained by incubating cells with streptavidin-functionalized Pdots in the absence of the primary biotinylated antibody. Cell fixation was performed by immersing the cells in 500 μl fixing buffer (1 × PBS, 2 mM EDTA, 1% BSA, 1% paraformaldehyde) for at least 15 min. Finally, the fixed cells were extracted by centrifugation and then redispersed in 500 μl 1 × PBS buffer.

### Cell labelling for fluorescence imaging

Cells were cultured in Petri dishes until ∼80% confluency was reached, then were gently washed with labelling buffer (1 × PBS, 2 mM EDTA, 1% BSA) and incubated with 0.6 μl of 0.5 mg ml^−1^ primary biotinylated anti-human CD326 (EpCAM) antibody (BioLegend, cat. #324216) in 100 μl of labelling buffer in the dark at room temperature for 30 min. Then, the cells were washed twice with labelling buffer to remove excess antibody, after which the cells were incubated with ∼5.0 nM streptavidin-functionalized Pdots in 0.2 ml of BlockAid buffer for 30 min in the dark at room temperature. The Pdot-labelled cells were washed twice to remove the excess Pdots.

### Flow cytometry

The flow cytometry measurements were performed on samples with ∼10^6^ fixed cells in 500 μl labelling buffer. The FACS CantoII (BD Bioscience) was used. Cells flowing into the detection chamber were irradiated with a 488-nm beam. The side- and forward-scattered light and excited fluorescence were collected by photomultiplier arrays. The fluorescence signal was collected in the PE channel, where the fluorescence was filtered by a 556-nm long-pass and a 585/42 nm band-pass filter. Representative populations of cells were chosen by selection of an appropriate gate. Detection of cell fluorescence was continued until 10^4^ events had been collected in the active gate. Data analysis was carried out by FlowJo Software (Tree Star, Inc., Ashland, OR, USA).

### Cellular imaging and painting

Cellular imaging was performed by an in-house fluorescence microscope with a 488-nm laser source under ambient conditions. A 633-nm laser was focused to a spot size of 10 μm in diameter for single-cell painting. A red light-emitting diode (M625L3, 1,000 mA) purchased from Thorlabs (Newton, NJ, USA) was used for wide-field illumination and cell painting. The photobleaching experiment was performed with a fluorescence confocal microscope (Zeiss LSM 510). The green and red dyes labelled on cells were excited by a 488-nm Argon laser and a 633-nm He–Ne laser, respectively. The 633-nm He–Ne laser (30 mW) was also used to photobleach the red dyes. The fluorescence signal of green and red channels was collected with 515/40-nm and 675/20-nm band-pass filters, respectively. A plan-Apochromat × 63/1.40 oil differential interference contrast objective lens was used for cellular surface imaging.

### Cell sorting via eDAR

The sorting process occurred in a microfluidic chip, which included a sorting area and a filtration unit. The sorting area contained a sample inlet channel with a section area of 150 × 50 μm. There were four other channels with 50 × 200 μm for injecting buffer and passing cells. The filtration unit was constructed with a 5 × 5 μm slit-filter to capture cells (for example, MCF-7 with a diameter ∼25 μm). The microfluidic chip was fabricated by one-step replica moudling into polydimethylsiloxane under a patterned silicon master and then sealed to a glass substrate via plasma oxidation. The Pdot-labelled MCF-7 cells in 1 × PBS with 10 wt % BSA were injected into the microfluidic chip by a syringe pump. Isoton from Beckman Coulter Inc. (Chino, CA, USA) was used as the buffer. The fluorescence signal of cells excited by a 488-nm laser beam was collected by fiber-coupled avalanche photodiodes from Excelitas Technologies (Waltham, MA, USA) with a filter of 570/20 nm. The sorting process of eDAR was automatically controlled by an in-house LabVIEW (National Instruments, Austin, TX, USA) script and a field-programmable gate array device built in-house. The sorting threshold was set based on the signal discrepancy between ON-state and OFF-state cells. The hydrodynamic sorting was controlled by a solenoid (INKA1226212H) purchased from the Lee Co. (Westbrook, CT, USA).

### DNA sequencing

Cells that were washed once in 1 × PBS were resuspended in a small volume of 1 × PBS to obtain a concentration of 1–100 cells per μl. Lysis was performed by incubating 5 μl of cell suspension and 6 μl nuclease-free water (Thermo Scientific, Marietta, OH, USA) at 99 °C for 10 min. A PCR master mix was then added to each lysate to obtain a final PCR mix containing 1 × Universal iTaq Supermix (Bio-Rad, Hercules, CA, USA) and 320 nM each of forward and reverse primer. The wild-type control set contained 5 ng purified human DNA. Primers ordered from Integrated DNA Technologies (Coralville, IA, USA) were using the sequence, forward, 5′-GGG AAA AAT ATG ACA AAG AAA GCT A-3′, and reverse, 5′-TCC ATT TTA GCA CTT ACC TGT GAC-3′. PCR conditions were 95 °C for 2 min followed by 45 cycles of 95 °C for 15 s, 57 °C for 45 s and 72 °C for 20 s. Agarose gel electrophoresis confirmed the presence of a single PCR product. Products were purified using MinElute columns (Qiagen, Redwood, CA, USA) and analysed using a Nanodrop 2000 to determine concentration and purity. Purified products were subjected to 10 μl cycle sequencing reaction using BigDye v3.1 (Life Technologies, Carlsbad, CA, USA) by a standard protocol with sequencing primer 5′-TAG CTA GAG ACA ATG AAT TAA GGG AAA-3′. Sanger sequencing was performed on an Applied Biosystems 3130xl Genetic Analyzer and data were analysed using MacVector with Assembler version 11.1.2 (MacVector, Inc., Cary, NC, USA).

### mRNA extraction and analysis

The sorted cell suspension was collected in an Eppendorf tube. Cells were counted after sorting using a hemocytometer. mRNA was extracted and purified using the PicoPure RNA Isolation Kit (Thermo Fisher Scientific, Grand Island, NY, USA) according to the manufacturer's protocol (https://www.thermofisher.com/order/catalog/product/KIT0204). The optional DNase treatment was performed according to the protocol using the RNase-Free DNase Set (Qiagen). All mRNA samples were quantified and assessed for integrity using an Agilent 2200 TapeStation. Concentrations of mRNA obtained from TapeStation analysis were compared with cell counts before mRNA isolation to determine the average mRNA recovery per cell.

### Genetically modified mice and animal care

All animal studies were approved by the Institutional Animal Care and Use Committee of the Fred Hutchinson Cancer Research Center. Tumours were obtained from the *Kras*^*LSL*-*G12D/+*^*;Trp53*^*LSL*-*R172H/+*^*;Pdx-1 Cre* (*KPC*) genetically engineered mouse model (male, 6.5 months of age) of PDA^45^. Animals were killed and tumours were removed and flash-frozen in optimal cutting temperature (OCT) compound (Tissue-Tek).

### Tissue-slice treatments

Blocked tissues were cryosectioned to 50-μm thickness via a Leica CM 3050S cryostats from Leica Biosystems (Buffalo Grove, IL, USA) and mounted on poly-L-lysine-coated microscope slides. The tissue slices were kept in −80 °C until use.

The 50-μm tissue slice was gently washed by 1 × PBS at least three times, then incubated with 5 μg biotinylated anti-mouse EpCAM (BioLegend, cat. #118203) in 0.5 ml 1 × PBS for 2 h. Then, the slice was gently washed three times with 1 × PBS to remove excess antibody, after which the slice was incubated with ∼5.0 nM streptavidin-functionalized Pdots in 0.5 ml of BlockAid buffer for 2 h in the dark at room temperature. The Pdot-labelled tissue slice was gently washed three times with 1 × PBS to remove the excess Pdots. Nuclear staining was carried out by immersing the tissue slice in DAPI (purchased from Thermo Fisher Scientific) solution (1 μM in 1 × PBS) for 1 min.

The painting process of cells in the tissue slice was the same as that described in the section Cellular imaging and painting.

The dissociation of the tissue slice into single cells was carried out via a commercial kit, Minute Single Cell Isolation Kit, purchased from Invent Biotechnologies, Inc. (Plymouth, MN, USA). The protocol is available at https://www.inventbiotech.com/single-cell-isolation-kit.php. The tissue slice was incubated with the dissociation buffer for non-fixed tissues. After several times of grinding by a plastic rod and incubation for 2–3 min, the solution was sent through a filter via centrifugation to collect the dissociated single cells.

## Additional information

**How to cite this article:** Kuo, C.-T. *et al*. Optical painting and fluorescence activated sorting of single adherent cells labelled with photoswitchable Pdots. *Nat. Commun.* 7:11468 doi: 10.1038/ncomms11468 (2016).

## Supplementary Material

Supplementary Information Supplementary Figures 1-13 and Supplementary References
