# Supplementary material for: Optical painting and fluorescence activated sorting of single adherent cells labelled with photoswitchable Pdots
Source: Nat Commun. 2016 Apr 27;7:11468. doi: 10.1038/ncomms11468 (PMC4853474; doi:10.1038/ncomms11468)
Supplement: Supplementary Information — Supplementary Figures 1-13 and Supplementary References [file ncomms11468-s1.pdf]

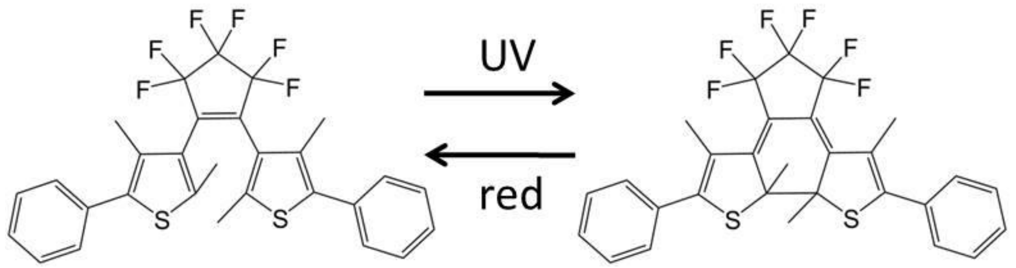

Supplementary Figure 1: Photochromic transition of BTE (1,2-bis(2,4-dimethyl-5-phenyl-3-thienyl)-3,3,4,4,5,5,-hexafluoro-1-cyclopentene) between the open-ring and closed-ring forms.

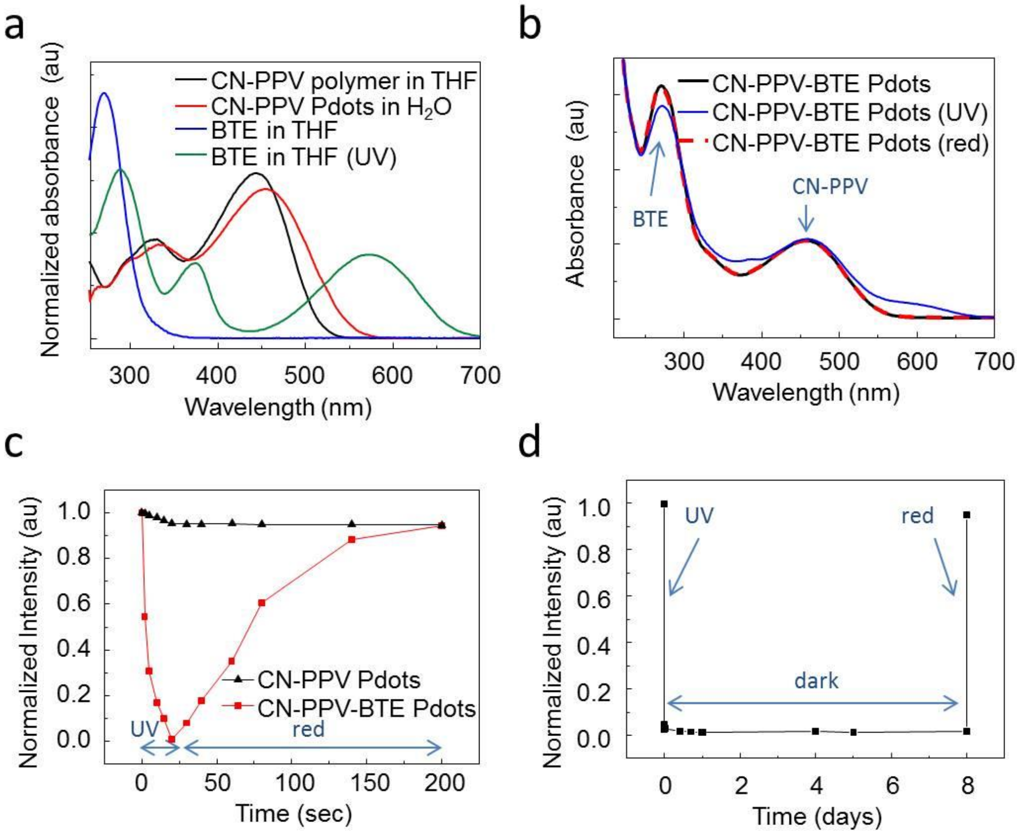

Supplementary Figure 2: Characterization of photoswitching of CN-PPV-BTE Pdots. (**a**) The UV/vis absorption spectra of CN-PPV and BTE both at 10 ppm dissolved in either THF or H_2O; “BTE in THF (UV)” was the absorption spectrum of BTE after it had been exposed to UV illumination. (**b**) UV/vis absorption spectra of CN-PPV-BTE Pdots before and after photoswitching; “CN-PPV-BTE” denotes before any light irradiation; “CN-PPV-BTE (UV)” denotes after illumination with UV light; “CNN-PPV-BTE (red)” denotes after subsequent illumination with UV and red lights. Comparing (a) and (b), we would find that two absorption peaks at 270 nm and 450 nm from the CN-PPV-BTE Pdots were attributed to BTE and CN-PPV, respectively. When Pdots solution was irradiated with UV light, the absorbance at 270 nm decreased and two small shoulders at around 375 nm and 580 nm simultaneously appeared, indicative of the conversion of doped BTE from the open-ring to the closed-ring form. The spectrum would recover after irradiation of the bulk sample with red light, indicative of the regeneration of the open-ring BTE. (**c**) Photoswitching kinetics of CN-PPV and CN-PPV-BTE Pdots in a bulk sample as reported by monitoring the fluorescence at 590 nm (excitation at 450 nm). (**d**) Thermal stability of CN-PPV-BTE Pdots; normalized intensity was based on monitoring the fluorescence at 590 nm (excitation at 450 nm).

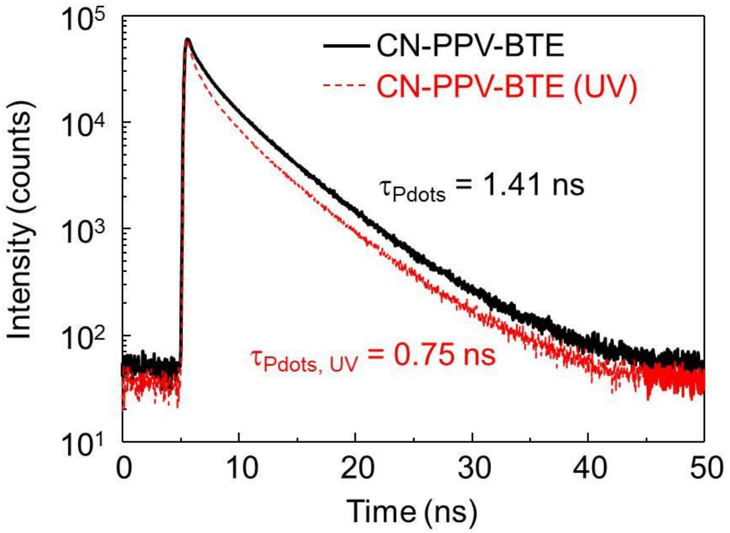

	A_1	t_1	A_2	t_2	τ $[(A_1t_1+A_2t_2)/A_1+A_2]$
CN-PPV-BTE	384600	0.356	164740	3.882	**1.413**
CN-PPV-BTE (UV)	632500	0.211	134240	3.294	**0.751**

Supplementary Figure 3: Fluorescence lifetime of CN-PPV-BTE Pdots in the ON and OFF state. The two time-resolved fluorescence decay curves indicate CN-PPV-BTE Pdots before and after irradiation with UV light. The fluorescence intensity was recorded as a function of time. The lifetime τ was obtained by fitting the decay curve with a two-phase decay model in which the outcome was the sum of a fast and slow exponential decay. The equation is $I(t) = A_1\exp(-t/t_1) + A_2\exp(-t/t_2)$, where I(t) indicates the fluorescence intensity at time t. The lifetime reported herein was calculated as a weighted arithmetic mean[1] $(A_1*t_1+A_2*t_2)/(A_1+A_2)$. The signal was collected above 490 nm upon excitation at 470 nm. The shorter lifetime of UV-irradiated CN-PPV-BTE Pdots (0.75 ns) compared to that of initial Pdots (1.41 ns) confirmed the presence of energy transfer from CN-PPV to BTE.

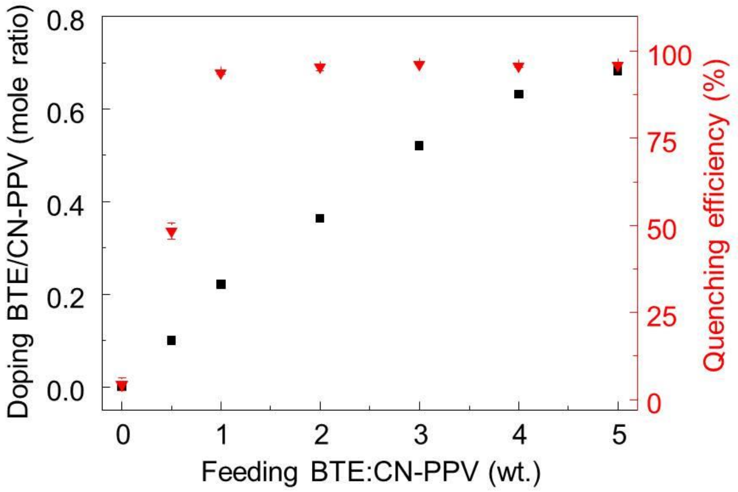

Supplementary Figure 4: Quenching efficiency of Pdots as a function of the amount of doped BTE. The doping ratio (by mole) and fluorescence quenching efficiency of CN-PPV-BTE Pdots as a function of feeding ratio (by weight) of BTE to CN-PPV. The doping ratio increased with the feeding ratio until 0.75 (BTE/CN-PPV, by mole). The quenching efficiency, defined as 1 − (OFF/ON ratio), increased with the increasing feeding ratio and achieved the maximum at a feeding ratio of 3 (BTE/CN-PPV, by weight). To optimize the doping ratio and the quenching efficiency, the feeding ratio of 3 was used for all experiments. The quenched state (OFF) was achieved by illumination of the CN-PPV-BTE Pdots with a common hand-held UV lamp (254 nm, 310 $\mu W\ cm^{-2}$) for 20 sec. The error bars represent the standard deviation from 3 separate experiments. All samples were with 20% (weight ratio) PS-PEG-COOH.

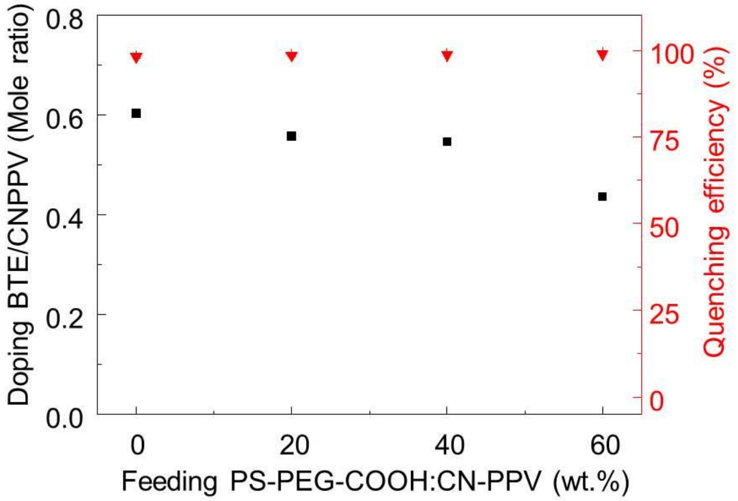

Supplementary Figure 5: Quenching efficiency of Pdots as a function of the amount of blended PS-PEG-COOH. The doping ratio (by mole) of BTE/CN-PPV and fluorescence quenching efficiency of CN-PPV-BTE Pdots as a function of feeding ratio (by percent weight) of PS-PEG-COOH to CN-PPV. The feeding ratio had no effect on the quenching efficiency. The PS-PEG-COOH:CN-PPV feeding ratio of 20% by weight was used for all the bioconjugation experiments. The quenched state (OFF) was achieved by illumination of the CN-PPV-BTE Pdots with a common hand-held UV lamp (254 nm, 310 μW cm^{-2}) for 20 sec. The error bars represent the standard deviation from 3 separate experiments. All samples were with a feeding ratio of 3 (BTE to CN-PPV, by weight).

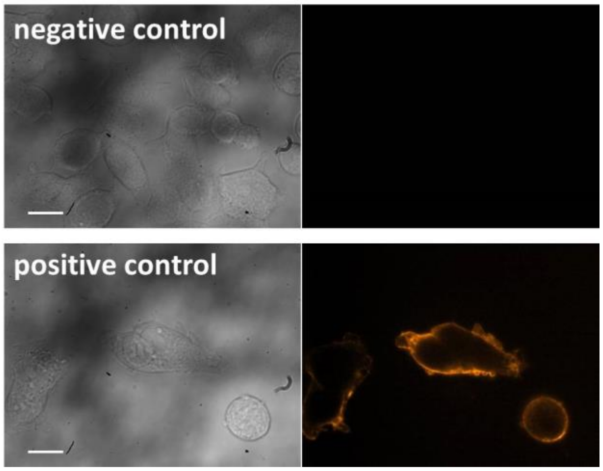

Supplementary Figure 6: Cellular imaging of the negative and positive control. The negative and positive control indicates samples where MCF-7 cells were incubated with CN-PPV-BTE-SA Pdots but in the absence (negative control) and presence (positive control) of biotinylated primary antibodies. The fluorescence signal was collected above 500 nm upon excitation at 488 nm. Scale bar, 20 μm.

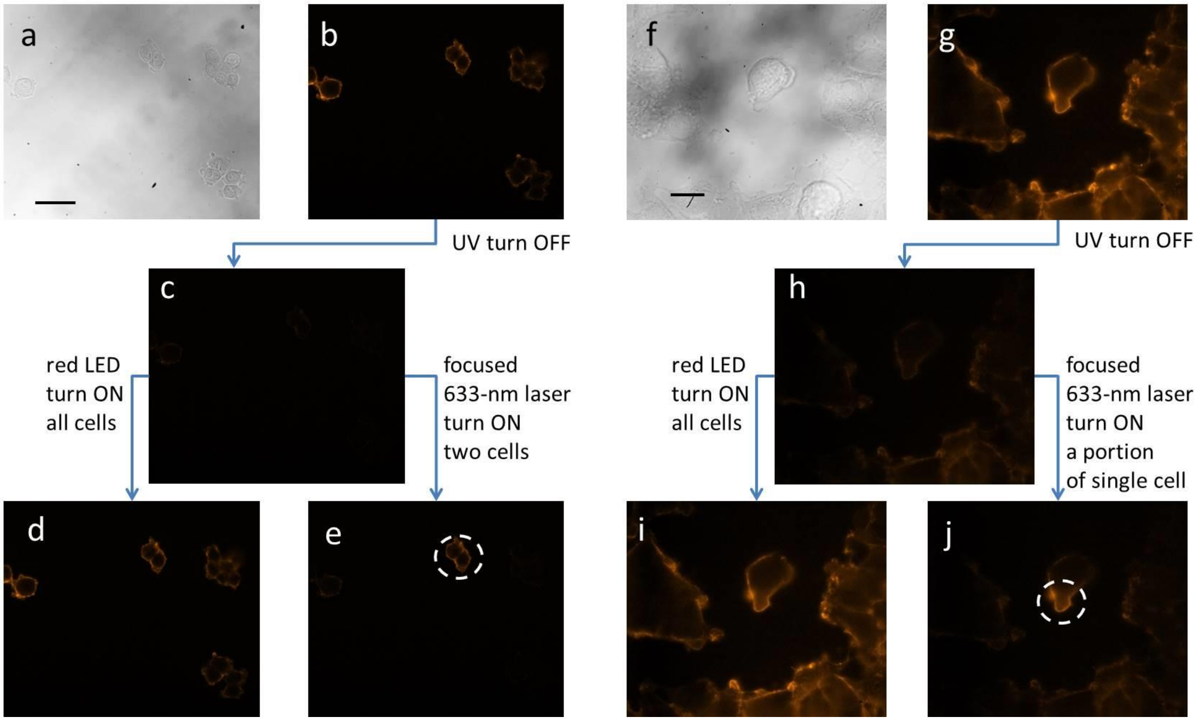

Supplementary Figure 7: Optical painting of multiple cells simultaneously and a portion of a single cell selectively. The cell-painting process that optically marked (**a-e**) two cells and (**f-j**) a portion of a single cell within multiple cells. Scale bars, 50 μm for **a** and 20 μm for **f**. All cells were in the dark (OFF) state when irradiated with UV light. Next, the selected cells or a portion of one cell underwent photoswitching and turned bright (ON state, indicated by a dashed circle) by the irradiation with a focused 633-nm laser. The UV treatment was carried out by a handheld UV lamp (254 nm, 310 μW cm^{-2}) for 20 sec. The red-light irradiation were performed with a focused 633-nm laser spot of a confocal microscope (~ 70 μW or 90 W cm^{-2}) for 10 sec or by a red LED light (625 nm, 1.5 mW cm^{-2}) for 3 min. The fluorescence signal was collected above 500 nm with a long-pass filter upon excitation at 488 nm.

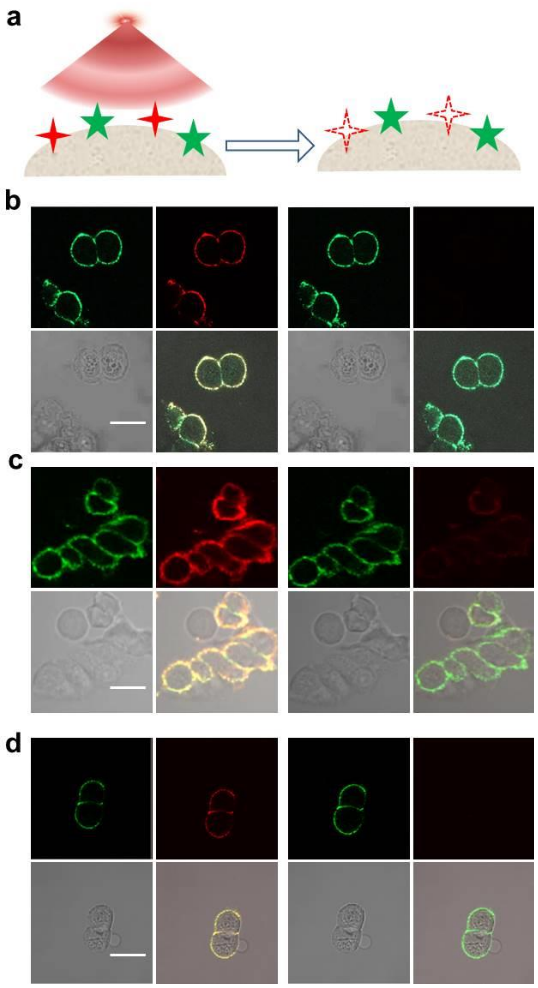

Supplementary Figure 8: Cell painting by selective photobleaching. (**a**) Schematic depiction of selective photobleaching. The red dye (cross) faded after irradiation while the green one (star) remained bright. The pairs of green-red fluorescent dyes labeled on cells were (**b**) FITC-APC, (**c**) Alexa 488-APC, and (**d**) Alexa 488-Alexa 647. All dyes were conjugated to primary antibody against EpCAM. In **b-d**, the upper left and upper right panels are obtained with the green and red fluorescence channels, respectively; the lower left panel is a bright-field image; the lower right panel is a merged image. After irradiation with a 633-nm laser (30 mW) for several minutes, the red fluorescence dimed while the green one remained bright. MCF-7 cells were used as examples. Scale bars, 25 μm.

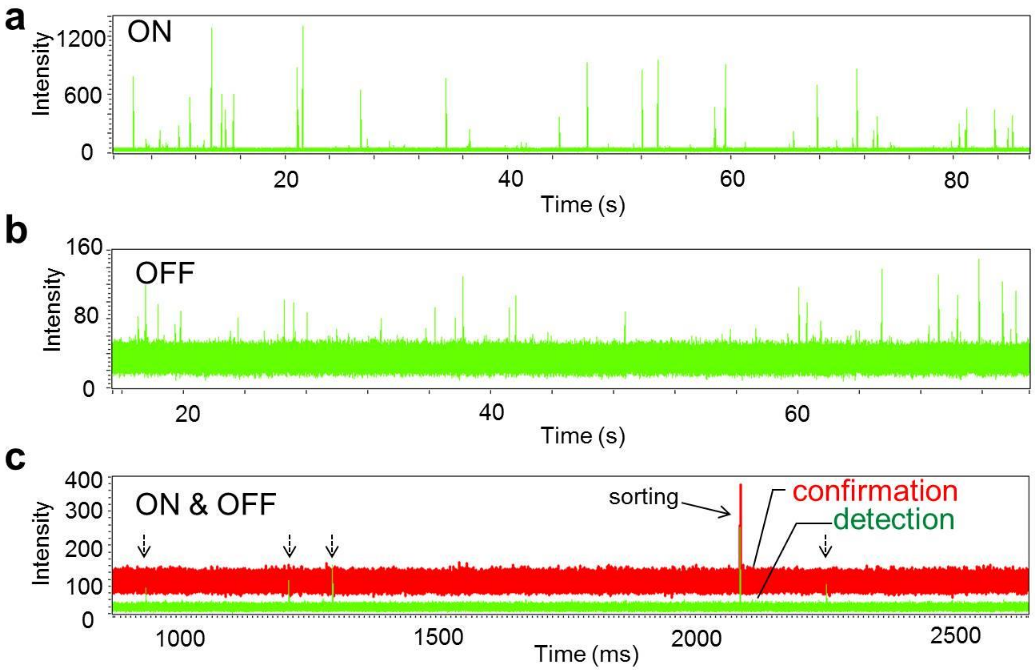

Supplementary Figure 9: Fluorescence signal from ON-state and OFF-state cells as detected by eDAR. Representative fluorescence intensity time traces obtained from (**a**) ON-state and (**b**) OFF-state MCF-7 cells recorded by the avalanche photodiode detector of our eDAR instrument[2,3]. (**c**) The segment of the detection and corresponding confirmation traces obtained from a sample in which ON-state cells were mixed with the OFF-state ones. The peak with the highest signal was present in both traces, indicating successful sorting of the ON-state cell. Other small peaks (dash arrows), which were from the OFF-state cells, could not trigger the sorting process so there was no corresponding peak appearing in the confirmation trace. The detection trace was recorded by a detector placed prior to the sorting junction, and the confirmation trace was by another detector placed at the collection channel after the sorting junction. The intensity threshold set for triggering the sorting system was 150 for detection trace.

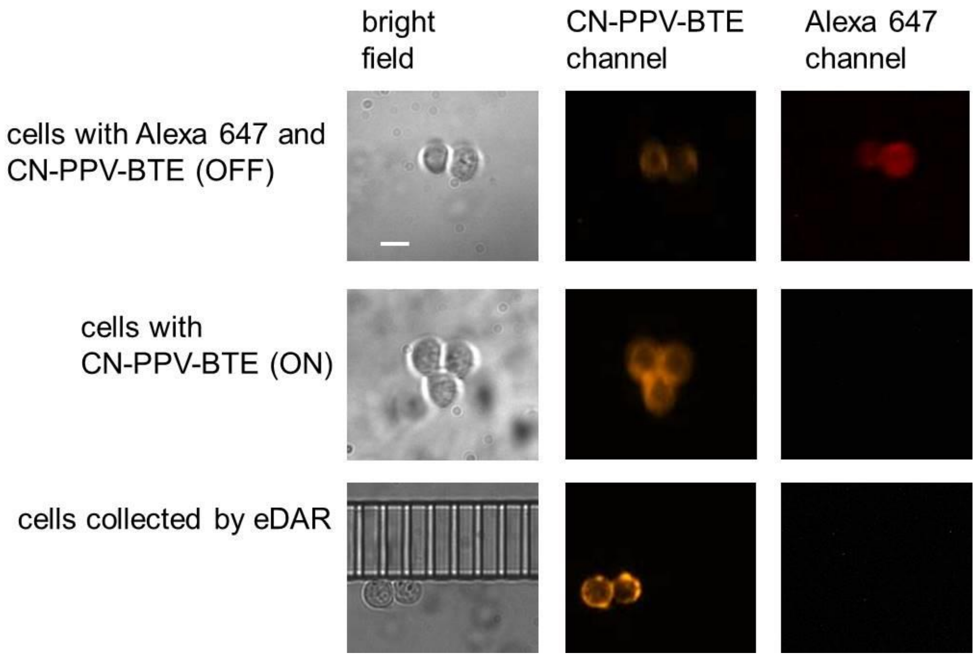

Supplementary Figure 10: No false positive cells were collected by eDAR. To mark OFF-state cells, they were further labeled with a dark-red dye (Alexa 647) so that the cells exhibited the fluorescence of Alexa 647 in addition to CN-PPV-BTE in the OFF state; the ON-state cells only exhibited the fluorescence of CN-PPV-BTE in the ON state without Alexa 647. After the mixed cells were sorted and collected by eDAR, all collected cells (~50) exhibited only the fluorescence of CN-PPV-BTE with no red fluorescence from Alexa 647. This indicates that the collected cells were all in the ON state and there was no accidental sorting of OFF-state cells. Scale bar, 20 μm. The cell images of the upper two rows were obtained when cells were extracted in a capillary tube used in cell spiking as described in our prior publication[4]. CN-PPV-BTE and Alexa 647 were excited by green and red lights from a Xe lamp and their fluorescence were separately collected with 570/20-nm and 675/30-nm band-pass filters, respectively.

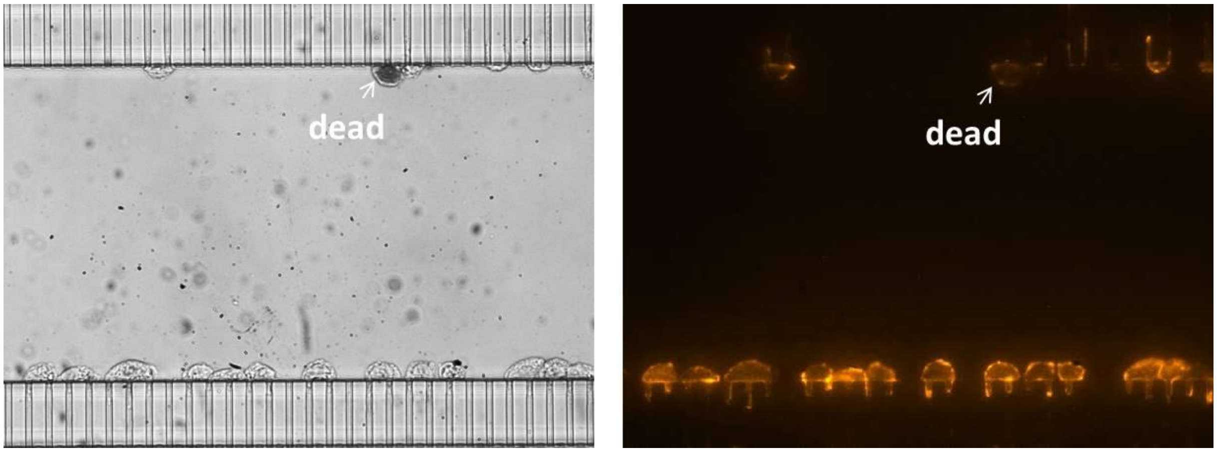

Supplementary Figure 11: Cells viability after the Pdot labeling, optical painting and sorting process. MCF-7 cells were labeled with OFF-form CN-PPV-BTE Pdots, painted by red light, then detached and dissociated by trypsinization, after which the painted cells were sorted and recovered by eDAR. These sorted and recovered cells were then checked for viability using the trypan blue assay, where dead cells would appear as dark blue in bright-field microscopy (pointed to by arrow in the left image) and live cells would remain clear. We found the viability was 90 ± 5 % (triplicate experiments) after the entire process of Pdot labeling, photo-painting, trypsinization and cell detachment, and eDAR sorting and recovery. The fluorescence image in the right panel shows all the recovered cells labeled with CN-PPV-BTE Pdots. The fluorescence image was obtained by excitation via a green light from a Xe lamp and by collecting the fluorescence with a 570/20-nm band-pass filter.

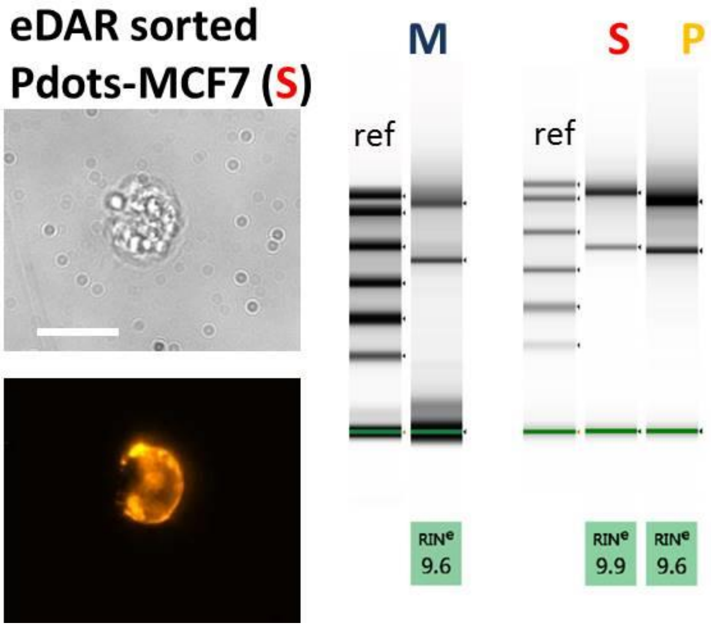

Supplementary Figure 12: mRNA analysis of cells collected through the optical painting and sorting process. The eDAR sorted MCF-7 cells (left panels) were tested for mRNA recovery and integrity using a TapeStation 2200 instrument (right panels). The CN-PPV fluorescence signal was collected by excitation via a green light from a Xe lamp and by collecting the fluorescence with a 570/20-nm band-pass filter. Scale bar, 20 μm. For the mRNA analysis (right panels), the gel image shows the separation profile of mRNA of the individual samples. “M”, “P”, and “S” indicate the untreated MCF-7 cells (M), Pdot-labeled MCF-7 cells (P), and Pdot-labeled then painted and eDAR sorted MCF-7 cells (S), respectively. The intensity of the band reflects the concentration of mRNA. The mRNA quality is presented as an RNA Integrity Number (RIN^e) value for each individual sample below the gel image (RIN^e is reported on a 1-10 scale, with 10 indicating the highest quality). Because the cell number of untreated MCF-7 (“M”) is much higher than the Pdot-labeled and eDAR-sorted samples (“P” and “S”), we used the Agilent R6K ScreenTape assay for sample “M” and the “High Sensitivity” R6K ScreenTape for “S” & “P” to precisely get the mRNA concentration. Therefore, there are two reference bands to calibrate the sample “M”, and “S” and “P”.

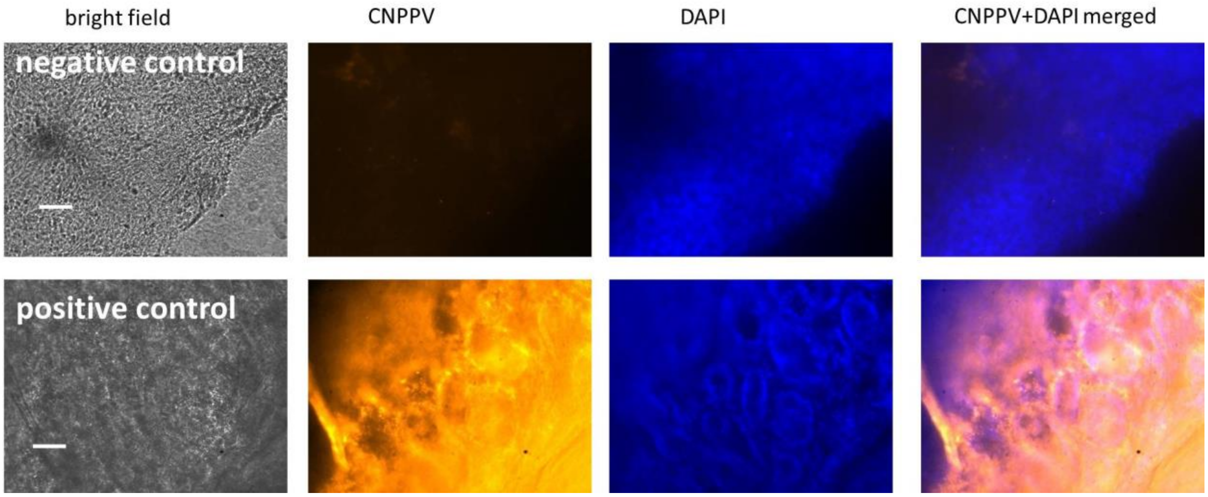

Supplementary Figure 13: Pancreas tumor tissue imaging of negative and positive controls. Pancreas tumor slices were incubated with CN-PPV-BTE-SA Pdots in the absence (negative control) or presence (positive control) of biotinylated primary antibodies. The CN-PPV and DAPI fluorescence signals were collected through 570/20-nm band-pass and 460/30-nm band-pass filters, respectively, upon excitation with a Xe lamp. Scale bar, 50 μm.

Supplementary References

1 Kishore, V. V. N. R., Narasimhan, K. L. & Periasamy, N. On the radiative lifetime, quantum yield and fluorescence decay of Alq in thin films. *Phys. Chem. Chem. Phys.* **5**, 1386-1391 (2003).

2 Schiro, P. G. *et al.* Sensitive and high-throughput isolation of rare cells from peripheral blood with ensemble-decision aliquot ranking. *Angew. Chem. Int. Ed.* **124**, 4696-4700 (2012).

3 Zhao, M. *et al.* New generation of ensemble-decision aliquot ranking based on simplified microfluidic components for large-capacity trapping of circulating tumor cells. *Anal. Chem.* **85**, 9671-9677 (2013).

4 Zhao, Y., Schiro, P. G., Kuo, J. S., Ng, L. & Chiu, D. T. Method for the accurate preparation of cell-spiking standards. *Anal. Chem.* **81**, 1285-1290 (2008).
